# Therapeutic progress in rosacea: targeting the neuro-vascular-immune triad

**DOI:** 10.3389/fimmu.2026.1876564

**Published:** 2026-06-22

**Authors:** Xingyin Yang, Wanqing Yang, Nuoran Chen, Dansheng Li, Yang Xu

**Affiliations:** 1 The Second Clinical Medical College of Nanjing Medical University, Nanjing, China; 2 Department of Dermatology, the First Affiliated Hospital with Nanjing Medical University, Nanjing, China; 3 Department of Dermatology, Beilun People’s Hospital, Ningbo, Zhejiang, China

**Keywords:** neurogenic inflammation, neuromodulation, neuro-vascular-immune triad, rosacea, targeted therapy

## Abstract

Rosacea is a chronic inflammatory dermatological condition predominantly affecting the facial region. Clinically, rosacea is characterized by paroxysmal flushing, persistent erythema, telangiectasia, papules, pustules, and ocular symptoms. Severe rosacea cases are frequently complicated by anxiety, depression, and sleep disturbances, imposing a heavy psychosocial burden and markedly impairing the quality of life of the patients. Abnormal activation of the neuro-vascular-immune triad has been reported as a crucial pathological mechanism of rosacea. Moreover, dysregulation within the central nervous system might exacerbate disease progression by modulating peripheral nerve activity and neuroendocrine homeostasis. In this review, we summarize the latest advancements in rosacea treatments targeting the neuro-vascular-immune triad, including γ-aminobutyric acid derivatives, antidepressants, anti-calcitonin gene-related peptide agents, physical neuromodulation (transcutaneous auricular vagus nerve and repetitive transcranial magnetic stimulations), botulinum toxin type A, and β-blockers. We provide a comprehensive analysis of their molecular mechanisms, clinical efficacy, and current limitations. While such therapies could specifically regulate different stages of the pathway, their evidence lacks large-scale randomized controlled trials and clarity regarding optimal dosing regimens and treatment durations. Future studies should strengthen basic investigations and clinical translation, explore combined therapeutic strategies, as well as develop personalized therapeutic strategies for the long-term effective control of rosacea.

## 1 Introduction

Rosacea is a chronic inflammatory skin disease commonly affecting the central area of the face. Its global prevalence is approximately 5.1% (1), with a relatively higher incidence in East Asia (2). Rosacea primarily affects individuals aged 45–60 years (3). Although the prevalence is higher among women, men tend to exhibit more pronounced nasal symptoms and higher-level overall disease severity (4). Clinically, rosacea is characterized by paroxysmal flushing, persistent erythema, and telangiectasia. Moreover, certain patients present with papules, pustules, and ocular symptoms (5). In severe cases, rosacea could contribute to psychological comorbidities such as anxiety and depression (6), thereby significantly impacting the quality of life of the patients.

Recent research advances suggest that abnormal activation of the neuro-vascular-immune triad may represent a key pathological feature associated with rosacea pathogenesis. The role of neural regulatory mechanisms in rosacea pathogenesis and progression has gained increasing recognition (7). Emerging therapeutic strategies targeting this triad have introduced novel perspectives for clinical intervention. In this review, we aimed to provide a comprehensive review of various treatment modalities for rosacea focusing on the neuro-vascular-immune triad, summarizing the underlying mechanisms, clinical efficacy, and limitations and allowing for valuable insights to inform both clinical practice and basic research on rosacea.

## 2 Materials and methods

### 2.1 Search strategy

This review follows the principles of narrative review research, systematically searching the PubMed, Web of Science, and China National Knowledge Infrastructure databases to select relevant studies published between 2015 and 2026. The search employed Boolean logic operators, with core search terms including rosacea, neurogenic inflammation, immune regulation, gabapentin, pregabalin, antidepressants, paroxetine, duloxetine, calcitonin gene-related peptide, erenumab, rimegepant, physical neuromodulation, transcutaneous auricular vagus nerve stimulation, repetitive transcranial magnetic stimulation, botulinum toxin, beta-blocker, carvedilol and propranolol. Additionally, the reference lists of included relevant studies were manually screened to supplement any literature missed in the database searches.

### 2.2 Inclusion criteria

Studies meeting the following criteria were included. First, clinical trials, observational studies, basic research, and systematic reviews focusing on rosacea were included. Second, studies investigating the mechanisms or efficacy of targeted therapies for the neuro-vascular-immune triad in rosacea were included. Third, published literature with clear research data and complete results was included.

### 2.3 Study selection processes

All retrieved results were imported into EndNote software to remove duplicate references. Two independent researchers screened the literature by reading titles and abstracts. In the first round, irrelevant and ineligible studies were excluded. The remaining studies were further evaluated through full-text reading. Discrepancies between the two researchers were resolved through discussion. If consensus could not be reached, a third researcher was consulted. Ultimately, eligible studies were included in this review.

### 2.4 Level of evidence assessment

We conducted an evidence level assessment for the clinical studies obtained from the above search to reflect methodological rigor. The evidence levels were based on the Oxford Centre for Evidence-Based Medicine (OCEBM) 2011 Levels of Evidence (8), ranging from Level 1 (highest quality) to Level 5 (lowest quality). The evidence levels corresponding to each study are summarized in **Table 1**.

**Table 1:** Summary of neuro-vascular-immune triad-targeting therapeutic strategies in rosacea treatment.

Category	Year	Author	Study type	Level of evidence	Participants	Treatment protocol	Therapeutic effect	Limitation
γ-aminobutyric acid (GABA) derivatives	2025	Ma et al. (36)	Multicenter, randomized, double-blind, placebo-controlled trial	Level 1	87 patients with refractory erythema of rosacea (73 completed)	Oral placebo, low-dose gabapentin (200 mg three times daily), high-dose gabapentin (300 mg three times daily) for 12 weeks with regular moisturizing	High-dose gabapentin significantly improved flushing and redness	Small sample size, lack of partial relapse data
	2025	Wei et al. (37)	Randomized controlled noninferiority trial	Level 1	315 patients with erythematotelangiectatic rosacea (ETR)	Gabapentin (300 mg three times daily), carvedilol (5 mg twice daily) for 12 weeks, followed by a 4-week drug-free period	Gabapentin was non-inferior to carvedilol in reducing clinical erythema assessment (CEA) score, rapidly alleviating flushing, migraine, and sleep disorders	Short follow-up period
	2024	Hurtado et al. (38)	Case report	Level 3	A 36-year-old woman with neurogenic rosacea (NR)	Pregabalin (150 mg daily) and duloxetine (90 mg daily) for 6 months, then combined with monthly intense pulsed light (IPL) for 3 months	Pregabalin and duloxetine markedly reduced pain and burning; combined therapy eliminated all symptoms and improved quality of life	Single case, limited generalizability
	2021	Kim et al. (39)	Multicenter retrospective case–control study	Level 3	17 NR patients, 106 ETR controls	NR: anticonvulsants (gabapentin/pregabalin) + antidepressants; ETR: conventional rosacea therapies	82.3% of NR patients improved with neuromodulators; 67.5% of ETR patients responded to conventional treatments	Small sample size, retrospective design, no standardized treatment protocol
Antidepressants	2023	Wang et al. (43)	Multicenter, randomized, double-blind, placebo-controlled trial	Level 1	112 patients with refractory erythema of rosacea (97 completed)	Paroxetine (25 mg daily), placebo for 12 weeks	Paroxetine significantly improved CEA success, flushing, burning sensation, and depression	Only a single dosage of paroxetine, short follow-up period
	2023	Chang et al. (44)	Randomized controlled trial	Level 1	90 patients with mild to moderate rosacea complicated with depression	Paroxetine hydrochloride (20–50 mg daily) + conventional treatment, conventional treatment for 3 months	Paroxetine significantly reduced self-rating anxiety scale (SAS) and self-rating depression Scale (SDS) scores and increased serum 5-hydroxytryptamine (5-HT) , Dopamine (DA) and Norepinephrine (NE) levels	Single-center study, short follow-up period
	2019	Wu et al. (45)	Randomized controlled trial	Level 1	128 patients with rosacea	Duloxetine hydrochloride (30–60 mg twice daily) + conventional treatment, conventional treatment for 12 weeks	Duloxetine significantly improved clinical effects, Hamilton Anxiety Rating Scale (HAMA)/Hamilton Depression Rating Scale (HAMD)/Dermatology Life Quality Index (DLQI) scores, and reduced recurrence rate	Single-center study, short follow-up period
	2022	Zhou et al. (46)	Randomized controlled trial	Level 1	89 patients with rosacea	Duloxetine hydrochloride (30–60 mg twice daily) + conventional treatment, conventional treatment for 12 weeks	Duloxetine reduced clinical symptom scores, SAS/SDS scores, and Th17/Treg ratio	Single-center study, short follow-up period, lack of quality of life assessment
Anti-calcitonin gene-related peptide (CGRP) agents	2023	Sia et al. (49)	Exploratory retrospective comparative case series	Level 3	13 patients with comorbid rosacea and migraine	CGRP monoclonal antibodies (galcanezumab, erenumab, and fremanezumab) combined with conventional treatment	CGRP monoclonal antibodies reduced overall rosacea severity, improved papules, pustules, erythema, flushing, and ocular symptoms	Single-center study, small sample size, short follow-up period, potential confounding by concurrent rosacea treatments
	2024	Wienholtz et al. (50)	Single-center, open-label, nonrandomized controlled trial	Level 2	30 patients with rosacea with persistent erythema or flushing (27 completed)	Subcutaneous erenumab (140 mg every 4 weeks) for 12 weeks	Erenumab significantly reduced moderate to extreme flushing days and moderate to severe erythema days; improved DLQI and RosaQoL scores	Open-label design without placebo control, small sample size, single-center study
	2025	Burke et al. (52)	Case report	Level 3	A 30-year-old woman with chronic migraine, refractory cystic acne, and polycystic ovary syndrome (PCOS)	Rimegepant for 4 weeks	Rimegepant cleared painful cystic acne lesions on the chin	Single case with no control group, lack of mechanistic verification
Physical Neuromodulation Therapy	2024	Wei et al. (54)	Case report	Level 3	A 22-year-old woman with refractory cutaneous dirt-adherent disease and ETR complicated with anxiety and depression	Transcutaneous auricular vagus nerve stimulation (taVNS) (30 Hz, 300 μs, 30 min daily) for 3 weeks + basic facial care	taVNS significantly reduced adherent crusts, erythema, flushing, anxiety, and depression	Single case without control group, unclear underlying mechanism
	2025	Li et al. (55)	Single-center, randomized, double-blind, sham-controlled trial	Level 1	72 ETR patients with CEA score ≥ 2	taVNS (30 Hz, 200 μs, 30 min daily), sham stimulation for 3 weeks	taVNS significantly decreased CEA, Patient Self-Assessment (PSA), and Global Flushing Severity Score (GFSS) scores; improved anxiety, depression, sleep disorders, migraine, and fatigue	Sham stimulation could not fully mimic active sensation, patient-reported bias
	2025	Song et al. (59)	Case series	Level 3	21 patients with ETR	Repetitive transcranial magnetic stimulation (rTMS) (low-frequency 1 Hz, targeting right dorsolateral prefrontal cortex [DLPFC]), 10 sessions within 2 weeks	rTMS significantly reduced GFSS, burning sensation, and anxiety; improved RosaQoL	No control group, small sample size
	2025	Zhang et al. (60)	Single-center, randomized, double-blind, sham-controlled trial	Level 1	77 patients with ETR	rTMS (high-frequency 10Hz, targeting left DLPFC, twice daily), sham stimulation for 2 weeks	rTMS significantly reduced CEA scores; improved PSA, GFSS, RosaQoL, anxiety, depression, and sleep	Short follow-up period, optimal stimulation parameters require further validation
Botulinum toxin	2022	Tong et al. (63)	Randomized, single-blind, split-face controlled trial	Level 1	22 patients with ETR	Botulinum toxin type A (BoNT-A) + broadband light (BBL), saline + BBL, 3 sessions at 1-month intervals	BoNT-A significantly reduced GFSS, erythema index, and transepidermal water loss (TEWL); increased skin hydration	Small sample size, imperfect control, unclear optimal dose and interval
	2025	Dağtaş et al. (64)	Prospective, randomized, double-blind, split-face controlled trial	Level 1	30 patients with ETR	Intradermal BoNT-A (15 U/cheek), saline, single session	BoNT-A significantly decreased CEA score, erythema index, and vascular density; improved patient self-assessment	Small sample size, 1-month short follow-up, patient-reported bias
Beta blockers	2022	Li et al. (68)	Randomized controlled trial	Level 1	145 patients with ETR	Oral carvedilol (5 mg twice daily), topical brimonidine for 10 weeks	Carvedilol significantly reduced erythema, flushing, burning, and stinging; improved anxiety, depression, and telangiectasia; better efficacy in patients under 30 years	Open-label design, high dropout rate, single-center, short follow-up period
	2015	Park et al. (70)	Non-randomized open-label trial	Level 2	78 patients with rosacea (32 ETR, 31 PPR)	Propranolol (10 mg 3 times daily), doxycycline (100 mg twice daily), combination for 12 weeks	All groups improved rosacea severity, combination therapy was most effective, propranolol favored flushing, doxycycline favored papules, and pustules	Non-randomized, non-blinded, no placebo, small sample size, unequal subtype distribution
	2017	Kwon et al. (71)	Case report	Level 3	A 37-year-old woman with refractory ETR	Oral propranolol + minocycline + tranexamic acid for 8 weeks	Propranolol improved refractory erythema, flushing, and telangiectasia within 1 week	Single case, no control group, limited generalizability of findings

## 3 Rosacea pathogenesis

### 3.1 Abnormal neuro-vascular-immune triad activation

A prominent pathological feature of rosacea is the abnormal activation of the neuro-vascular-immune triad, and its cascade amplification is considered to be associated with the development of core clinical manifestations such as flushing, persistent erythema, telangiectasia, and chronic inflammation. In genetically susceptible individuals, exposure to triggers (e.g., ultraviolet radiation, temperature fluctuations, and microbial stimuli) (9) may trigger the activation of transient receptor potential vanilloid subfamily (TRPV1 and TRPV4) and transient receptor potential ankyrin 1 (TRPA1) in the skin (10, 11). Moreover, cutaneous sensory nerve endings in rosacea release numerous neuropeptides, including pituitary adenylate cyclase−activating polypeptide (PACAP), calcitonin gene−related peptide (CGRP), substance P (SP), and vasoactive intestinal peptide (VIP). These neuropeptides induce vasodilation and angiogenesis, and trigger mast cell degranulation via Mas-related G protein-coupled receptor X2 (MRGPRX2), leading to the release of TNF−α, histamine, tryptase, proinflammatory cytokines, and other mediators, which further promote downstream inflammatory responses (12–15). Concurrently, Toll-like receptor 2 (TLR2) expression is upregulated in keratinocytes from patients with rosacea, thereby enhancing the production and release of kallikrein 5 (KLK5). KLK5 then converts the antimicrobial peptide precursor hCAP18 into its active form LL-37 (16, 17). Subsequently, LL-37 triggers the NF-κB signaling cascade and NLRP3 inflammasome, leading to enhanced pro-inflammatory cytokine (e.g., IL-1β and TNF-α) production (18). Furthermore, LL-37 facilitates mast cell degranulation via MRGPRX2, inducing the release of mediators such as histamine, tryptase, FGF2 and VEGF (19, 20). Histamine can increase vascular permeability through NO-dependent vasodilation, increased blood flow, and disruption of the vascular endothelial barrier (21). FGF, VEGF and other factors promote angiogenesis, which might correspond to the clinical manifestations of erythema and telangiectasia in rosacea (20). These mediators can exacerbate the release of neuropeptides from sensory nerve endings, further amplifying neurogenic inflammation (22). In addition, ultraviolet radiation can impair the skin’s ability to repair environmental damage through the vitamin D and its receptor (VDR) signaling pathway, further exacerbating the inflammatory response (23).

### 3.2 Central nervous system regulatory dysfunction

The relationship between rosacea and central nervous system regulatory dysfunction is complex, with mutual influence and comorbidity associations. On one hand, a cross-sectional survey conducted by the National Rosacea Society (NRS) in the United States involving 1, 675 patients showed that 90% of patients experienced decreased self-esteem and confidence due to impaired appearance, severely damaging their mental health (24). Neuroimaging studies using 18F-FDG PET/CT have allowed for identifying distinctive abnormalities in glucose metabolism within the cerebral cortex and limbic system of patients with rosacea, indicating that the primary brain regions responsible for emotional processing and autonomic nervous system regulation underwent functional reorganization (25). On the other hand, chronic stress caused by anxiety and depression activates TRP channels, amplifying neuroinflammatory responses in the skin and worsening symptoms such as flushing and erythema (26). A meta-analysis published in 2022 indicated that rosacea is strongly correlated with affective disorders (e.g., depression and anxiety) (6). Another meta-analysis showed that the prevalence of migraine is higher in this population, with the incidence nearly doubling compared to that in non-affected groups. Notably, both rosacea and migraine share a pathological basis centered on CGRP-mediated neurogenic inflammation and vascular hyperreactivity (27). Additionally, a retrospective cohort study indicates a significantly elevated risk of sleep disturbances among patients with rosacea, particularly women and those with dyslipidemia (28). However, there is still no direct evidence to clarify whether central nervous system abnormalities are inherent to rosacea itself or secondary to the anxiety and depression comorbid with rosacea.

Dysregulation of the hypothalamic-pituitary-adrenal (HPA) axis plays an important role in rosacea pathogenesis. Glucocorticoids (GCs), acting as the terminal effectors of the HPA axis, are crucial for maintaining cutaneous homeostasis. Nevertheless, chronic stress can perturb the HPA axis activity and impair its negative feedback loop, ultimately culminating in glucocorticoid resistance (29). Notably, skin possesses an independent cutaneous HPA-like axis, capable of autonomously synthesizing stress hormones such as corticotropin-releasing hormone (CRH) and cortisol (30). Phan et al. elucidated the important role of keratinocyte-derived GCs and cutaneous HPA-like axis in maintaining skin homeostasis through a mouse model study (31). CRH neurons stimulate the TRPV1 and cannabinoid type 1 (CB1) pathways, contributing to rosacea progression (32). Furthermore, CRH may act synergistically with cortisol to upregulate TLR2 expression, inducing CXCL8 release and subsequently promoting neutrophil chemotaxis and angiogenesis (26). In addition, CRH promotes mast cell degranulation, regulates IL-18 secretion in keratinocytes and IL-6 and IL-8 production in sebocytes, activates the MAPK and NF-κB signaling pathways, and thereby induces facial erythema and inflammation in patients with rosacea (33).

## 4 Diverse therapeutic strategies targeting the neuro-vascular-immune triad

Recently, numerous neuro-vascular-immune triad-targeting therapeutic agents and modalities have demonstrated clinical efficacy (**Table 1**). However, several aspects still require further exploration.

### 4.1 γ-aminobutyric acid (GABA) derivatives

GABA derivatives (e.g., gabapentin and pregabalin) act on the α2δ subunit of neuronal voltage-gated calcium channels, reducing calcium ion influx, thereby decreasing the release of neuropeptides like SP and inhibiting neurogenic inflammation (34). Gabapentin reportedly downregulates pro-inflammatory cytokines by suppressing NF-κB signaling pathway activation, and inhibits angiogenesis-associated protein expression, thereby suppressing neurogenic inflammation and vasodilation in rosacea (35).

Several clinical trials have confirmed the therapeutic efficacy of gabapentin. A multicenter, randomized, double-blind, placebo-controlled study (n = 87) revealed that high-dose gabapentin (300 mg thrice daily) treatment for 12 weeks significantly improved flushing and redness relative to both placebo and low-dose gabapentin (200 mg thrice daily) (36). In the same year, another randomized non-inferiority trial (n = 315) further corroborated that gabapentin was non-inferior to carvedilol (5 mg twice daily) in reducing erythema among patients with erythematotelangiectatic rosacea (ETR). Notably, gabapentin was significantly superior to carvedilol as well in improving sleep disorders and migraines, but its efficacy in alleviating anxiety and depression was weaker than that of carvedilol (37).

Pregabalin and gabapentin share a similar effector mechanism. A case report published in 2024 described a patient with neurogenic rosacea complicated by anxiety and depression. Treatment with 150 mg pregabalin and 90 mg duloxetine daily for 6 months reportedly reduced pain and burning sensations (38). Moreover, a multicenter retrospective study (n = 17) confirmed that 82.3% of patients treated with either pregabalin or gabapentin exhibited marked improvement in severe persistent erythema, burning, and stinging symptoms. The benefits were more prominent in refractory cases with comorbid anxiety and depression (39). Current evidence for pregabalin use in rosacea is mainly based on small-sample clinical studies. The optimal dosage and treatment duration remain to be further defined.

GABA derivatives are not conventionally used as first-line treatments for rosacea. Their use is selective and prioritized for refractory ETR patients with significant flushing, burning, and other sensory abnormalities, especially those with comorbid migraine or sleep disorders. Given that adverse reactions in the high-dose gabapentin group remain mild and self-limiting, a regimen of 300 mg thrice daily can be considered. However, for patients with poor tolerance, it is recommended to start at a low dose and gradually increase.

### 4.2 Antidepressants

Antidepressants represent a novel therapeutic approach for rosacea, particularly neurogenic rosacea, through mechanisms involving neurotransmitter homeostasis regulation, neurogenic inflammation suppression, and vasomotor function modulation (40).

Paroxetine, a selective serotonin reuptake inhibitor (SSRI), has been shown to reduce the levels of IL-1β, IL-6, and TNF-α, which may contribute to the improvement of facial erythema. It has also been found that the decrease in inflammatory response levels is related to the degree of improvement in depressive symptoms. Patients with a greater reduction in pro-inflammatory cytokine levels also experience a more significant improvement in the severity of depression (41, 42). A multicenter randomized controlled trial (n = 97) confirmed that 12-week paroxetine (25 mg daily) treatment significantly improved the success rate of clinical erythema assessment (CEA), flushing, and burning sensations while ameliorating comorbid depressive symptoms (43). However, another randomized controlled trial (n = 90) indicated that paroxetine combined with conventional therapy failed to enhance overall cutaneous efficacy in mild-to-moderate rosacea with depression, though it effectively reduced anxiety and depression scores and increased monoamine neurotransmitter levels (44).

Duloxetine, a serotonin and norepinephrine reuptake inhibitor (SNRI), is frequently combined with conventional therapies for rosacea management. Two single-center RCTs (n=128 and n=89) confirmed that adding duloxetine to doxycycline and topical metronidazole for 12 weeks significantly improved papulopustular, erythema and pruritus scores, along with alleviating anxiety and depression (45, 46). Based on the above clinical trial results, it can be reasonably inferred that antidepressants are more suitable for patients with moderate-to-severe rosacea accompanied by anxiety and depression. In the study by Chang et al. (44), the benefit to skin symptoms was not significant, which may be attributed to the inclusion of patients with mild-to-moderate conditions and differences in the study design involving combined conventional treatment. Given the regulatory role of psychobiological factors in the progression of rosacea, further evaluation of the long-term efficacy, safety, and potential drug interactions of antidepressant treatment is needed.

Yet, there is currently no evidence confirming whether antidepressants treat rosacea by directly inhibiting cutaneous inflammation; their presumed mechanisms remain inferred solely from available clinical outcomes.

### 4.3 Anti-CGRP agents

Aberrant activation of the CGRP signaling pathway plays a crucial role in both rosacea and migraine pathogenesis. A cross-sectional case-control study demonstrated elevated plasma CGRP concentrations in patients with rosacea relative to healthy controls (47). Consequently, targeting CGRP has become a prominent focus in clinical management. The drugs mainly include two categories: anti-CGRP receptor monoclonal antibodies (such as erenumab) and small molecule CGRP receptor antagonists (such as Rimegepant) (48).

A retrospective case series (n = 13) of patients with rosacea and comorbid migraine revealed that CGRP monoclonal antibody treatment can improve papulopustular and erythema flushing symptoms in 54% of patients (49). Furthermore, a non-randomized controlled trial (n = 30) confirmed that 12-week subcutaneous erenumab (140 mg every 4 weeks) significantly decreased the number of days with moderate-to-severe facial flushing and erythema in patients with rosacea. However, 83% of participants reported at least one adverse event, predominantly mild-to-moderate constipation (33%) (50). These findings provide preliminary evidence supporting the therapeutic potential of erenumab in rosacea. Larger randomized controlled future trials would be required to better determine its long-term clinical outcomes and safety profile.

In terms of small-molecule CGRP receptor antagonists, animal model studies have demonstrated that oral rimegepant mitigates capsaicin-induced rosacea-like dermatitis by inhibiting the nerve-CGRP-γδT cell axis (51). Burke et al. reported a case in which a patient with refractory inflammatory dermatosis and comorbid migraine experienced significant improvement in skin lesions within 4 weeks after treatment with rimegepant (52). The clinical application of anti-CGRP agents is still in the exploratory stage. However, based on previous reports in basic research and migraine treatment, anti-CGRP agents show great potential. Larger-scale clinical trials are needed in the future to further evaluate their efficacy and safety.

### 4.4 Physical neuromodulation therapy

Neuromodulation is a novel therapeutic strategy that uses physical techniques to modulate nervous system functions. Transcutaneous auricular vagus nerve stimulation (taVNS) and repetitive transcranial magnetic stimulation (rTMS) are noninvasive techniques that show potential value in rosacea treatment. taVNS targets the vagus nerve in the auricular concha and improves central nervous system disorders such as epilepsy, depression, and insomnia (53). In 2024, Wei et al. reported a case of refractory cutaneous dirt adherent disease complicated with ETR that exhibited significantly reduced adherent crusts, erythema, and flushing after 3 weeks of taVNS treatment (30 Hz frequency, 300 μs pulse width, 30 min daily). The symptoms of anxiety and depression were also markedly alleviated (54). A subsequent randomized controlled trial (n = 72) further confirmed that the taVNS group (30 Hz frequency, 200 μs pulse width, 30 min daily) showed better improvements in facial erythema, flushing, and comorbidities like anxiety, depression, and sleep disorders (55).

rTMS has been widely used in the treatment of neurological and psychiatric disorders. Different frequencies of rTMS have different mechanisms of action and indications (56). 1 Hz low-frequency rTMS targeting the right dorsolateral prefrontal cortex (DLPFC) has been shown to alleviate symptoms of autonomic dysfunction like anxiety and insomnia (57). 10 Hz high-frequency rTMS stimulation of the left DLPFC is considered one of the most evidence-based methods for treating depression (58). The parameters of rTMS used for treating rosacea follow the effective protocols applied in psychiatry for anxiety and depression. A single-arm case series (n = 21) reported that low-frequency (1 Hz) right DLPFC rTMS improved flushing, burning, and anxiety after 10 sessions, with a 12-week follow-up (59). In the same year, a randomized controlled trial (n = 77) demonstrated that high-frequency (10 Hz) left DLPFC rTMS significantly ameliorated facial erythema, flushing, and associated comorbidities like depression and sleep disorders (60). These two studies indicate that both low-frequency and high-frequency rTMs are effective in treating rosacea, and the choice of frequency can be comprehensively considered based on the patient’s psychiatric comorbidities.

Both taVNS and rTMS improve rosacea cutaneous manifestations and alleviate comorbid neuropsychological symptoms, yet no clinical data support their combined use in rosacea. Notably, a clinical trial protocol has proposed synergistic benefits of their combination in mild cognitive impairment (MCI), suggesting the theoretical feasibility of this approach (61). Therefore, future research should investigate the clinical efficacy of combined taVNS and rTMS for rosacea.

### 4.5 Vascular-targeted therapy

#### 4.5.1 Traditional vascular-targeted therapy

The efficacy of botulinum toxin in rosacea has been well demonstrated. Beyond its traditional role in inhibiting neurotransmitter release, botulinum toxin type A (BoNT/A) can facilitate LL37-mediated internalization of the MRGPRX2 receptor, reducing the number of functional MRGPRX2 receptors on the cell membrane surface. This causes mast cells to become desensitized to LL37 stimulation, decreasing the synthesis and secretion of vascular endothelial growth factor (VEGF), thereby inhibiting angiogenesis and alleviating symptoms such as erythema and capillary dilation (62). A randomized split-face controlled trial (n = 22) revealed that intradermal BoNT/A (10–15 U per cheek) combined with broadband light (BBL) outperformed BBL monotherapy in improving erythema, flushing, and skin barrier function (63). Similarly, another randomized double-blind controlled trial (n = 30) demonstrated that intradermal BoNT-A (15 U per cheek) alone significantly reduced erythema index and vascular density in ETR patients at 1-month follow-up (64). The expert consensus recommends a total dose of 30 to 100 units of type A botulinum toxin per treatment, with no more than 50 units on one side of the cheek (65).

Beta blockers can mitigate erythema by blocking beta adrenergic receptors on cutaneous vascular smooth muscle to induce vasoconstriction. Additionally, these agents act on myocardial beta adrenergic receptors to reduce heart rate and alleviate anxiety (66). Carvedilol and propranolol are the beta blockers commonly used for the treatment of rosacea. Carvedilol is a nonselective beta blocker with newly discovered anti-inflammatory effects that reduce the secretion of pro-inflammatory cytokines such as TNF-α and IL-6 by inhibiting macrophage TLR2 expression and decreasing KLK5 and LL-37 levels (67). A randomized controlled trial (n = 145) demonstrated that oral carvedilol (5 mg twice daily) for 10 weeks significantly reduced CEA and PSA scores while improving anxiety and depression in patients with ETR (68). Furthermore, Chen et al. have developed a thermosensitive hydrogel delivery system that effectively minimized the side effects of oral carvedilol and achieved favorable therapeutic outcomes through targeted topical application in a rosacea-like mouse model (69). Meanwhile, clinical evidence for propranolol primarily derives from case reports and small sample trials. A prospective cohort study (n = 63) indicated that a 12-week treatment with propranolol (10 mg thrice daily) was significantly superior to doxycycline monotherapy in alleviating flushing (70). Furthermore, Kwon et al. described the case of a 37-year-old patient with severe rosacea who exhibited marked improvement in erythema and subjective symptoms after 1 week of combined treatment with propranolol, minocycline, and tranexamic acid (71).

**Figure 1:**
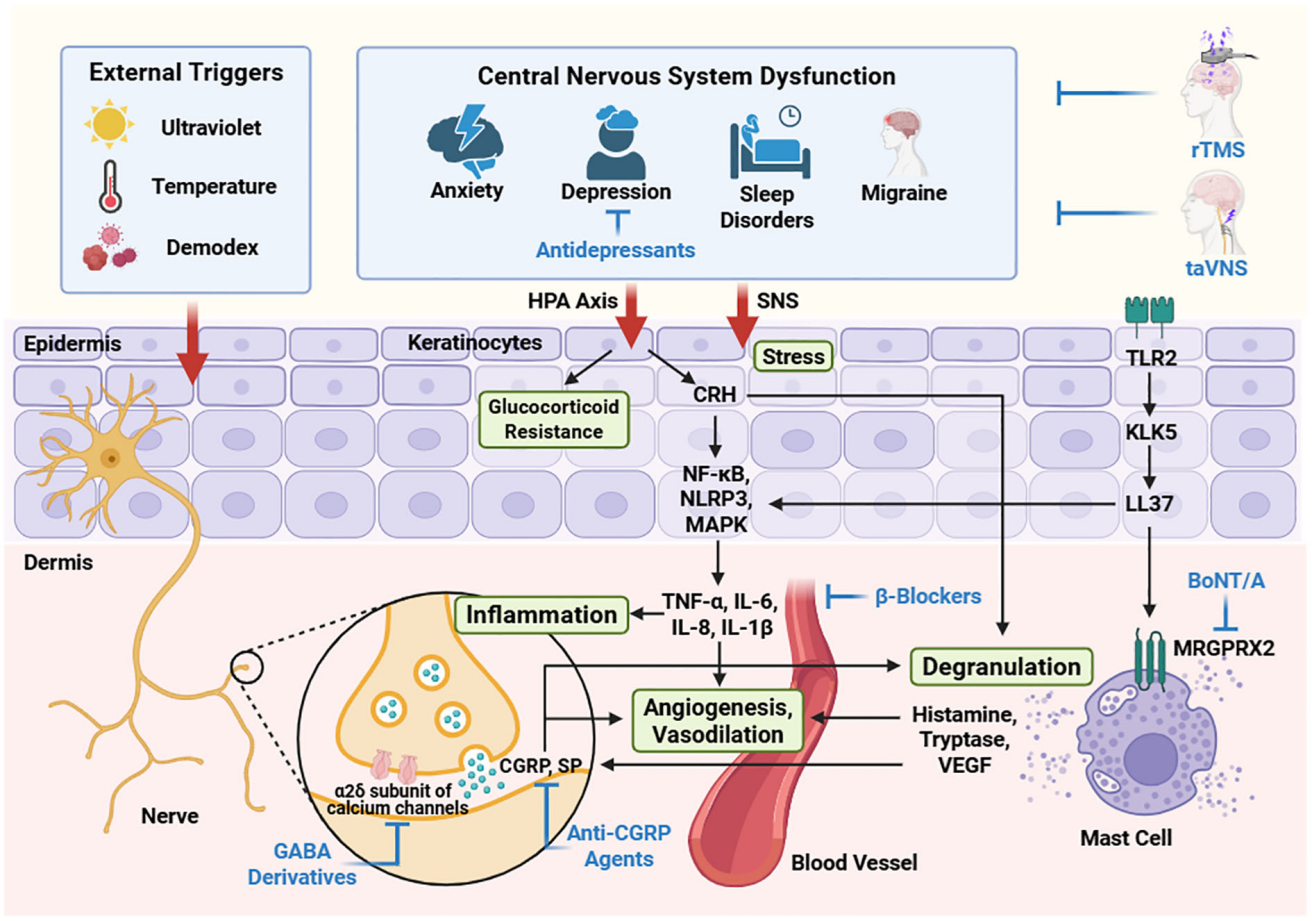
Rosacea pathogenesis and related targeted therapeutic strategies within the neuro-vascular-immune triad. External triggers stimulate the epidermis and activate the TLR2/KLK5/LL-37 signaling pathway in keratinocytes. This activation subsequently triggers the NF-κB signaling cascade and NLRP3 inflammasome, leading to pro-inflammatory cytokine production. Moreover, LL-37 facilitates mast cell degranulation via the Mas-related G protein-coupled receptor X2 (MRGPRX2) receptor, resulting in histamine, tryptase, and vascular endothelial growth factor (VEGF) release, strongly promoting angiogenesis. In the peripheral nervous system, these triggers activate TRPA1 and TRPV1 channels on sensory nerve endings, thereby provoking the release of neuropeptides such as calcitonin gene-related peptide (CGRP) and substance P (SP). Furthermore, central nervous system dysregulation drives rosacea progression through hypothalamic-pituitary-adrenal (HPA) axis dysfunction, chronic stress and affective disorders, which modulate peripheral nerve activity and neuroendocrine homeostasis. Various therapeutic strategies specifically target this neuro-vascular-immune triad. Physical neuromodulation therapies, including transcutaneous auricular vagus nerve stimulation (taVNS) and repetitive transcranial magnetic stimulation (rTMS), modulate central nervous system dysfunction. Antidepressants are used to mitigate depression and central neuroendocrine dysregulation. In the peripheral pathways, γ-aminobutyric acid (GABA) derivatives inhibit the α2δ subunit of neuronal voltage-gated calcium channels. Anti-CGRP agents block vasoactive neuropeptide signaling. β-blockers act on blood vessels to mitigate vasodilation and erythema. Botulinum toxin type A (BoNT/A) suppresses angiogenesis and flushing by inhibiting LL37-mediated mast cell MRGPRX2 activation.

**Figure 1** illustrates the therapeutic mechanisms of the aforementioned agents and modalities. With continuous research progress, new related therapeutic drugs and methods are constantly emerging.

#### 4.5.2 Emerging metabolic-vascular target

A recent study integrating basic research and animal model experiments, with small-sample human correlation analysis published in *Cell* reveals that α-ketoglutarate (α-KG) ameliorates abnormal vasodilation by OXGR1 receptor activation and upstream metabolic axis modulation. This discovery provides a highly promising direction for the treatment of rosacea. As an approved dietary supplement with established safety, α-KG topical gel formulations can quickly proceed to clinical validation and are suitable for patients with mild to moderate erythema. α-KG/OXGR1-targeted therapy can also be combined with existing anti-inflammatory and neuroregulatory drugs to achieve synergistic regulation of the neuro-vascular-immune triad, enhancing the efficacy in treating refractory rosacea. Future research still needs to further elucidate the pathogenesis of rosacea and conduct clinical trials to evaluate the long-term efficacy and safety of this new approach (72).

## 5 Discussion

Rosacea can be categorized according to its clinical presentations into four subtypes: erythematotelangiectatic rosacea (ETR), papulopustular rosacea (PPR), phymatous rosacea (PhR), and ocular rosacea (OR) (73). Among these, ETR is the primary group suitable for targeted neuro-vascular-immune triad therapies. Beta blockers are appropriate for ETR patients whose main symptoms are persistent erythema and paroxysmal flushing; blood pressure should be closely monitored during treatment, and withdrawal reactions should be noted. Although some controversy remains, several investigators regard neurogenic rosacea as a distinct subtype, characterized by prominent facial erythema refractory to conventional therapy, accompanied by severe burning and tingling sensations. In some patients, the severity of subjective symptoms (burning/tingling) far outweighs objective clinical signs. These symptoms are often managed with neuropsychiatric medications, and patients may present with or without comorbid neuropsychiatric conditions (e.g., depression, obsessive−compulsive disorder, essential tremor, migraine) (74). Conversely, other researchers consider neurogenic rosacea to represent a severe, treatment−refractory subset within ETR (75). Physical neuroregulation therapy, GABA derivatives and BoNT/A can be used for NR or ETR patients unresponsive to conventional treatments. For patients with anxiety and/or depression, antidepressant treatment can be prioritized. Although one clinical study has shown that erenumab can effectively alleviate erythema and flushing in ETR (50), more randomized, double-blind, controlled trials are still needed to confirm its clinical efficacy. Clinical evidence for anti-CGRP agents is still insufficient, and their use is recommended with caution in rosacea patients who also suffer from migraines.

The overall safety of targeting the neuro-vascular-immune triad for rosacea treatment is favorable, with adverse reactions mainly being mild and transient. The adverse reactions of β-blockers mainly include nausea and vomiting, dizziness, insomnia, and indigestion. The adverse reactions reported for gabapentin are commonly dizziness and drowsiness. Antidepressants have a relatively wider range of adverse reactions, commonly including dizziness, drowsiness, headache, and gastrointestinal reactions, and less commonly liver function abnormalities, muscle tremors, and tinnitus. The adverse reactions of the above drugs are mostly self-limiting and can be alleviated by gradual dose titration. Physical neuromodulation methods such as taVNS and rTMS have excellent safety profiles, with only transient local symptoms observed, such as mild headache and tinnitus, and no serious systemic complications. It should be noted that clinical trials conducted by Wienholtz et al. reported constipation as the most common adverse reaction to erenumab. More attention should be paid to the side effects reported in as high rates as 83% with newer agents such as erenumab (50). Type A botulinum toxin is associated only with mild injection site reactions; occasionally, transient facial muscle paralysis occurs due to drug diffusion, appearing on the 10th day after injection and completely resolving spontaneously by the 40th day without sequelae (64). Overall, data from the aforementioned clinical trials suggest that novel therapeutic approaches targeting the neuro-vascular-immune triad possess a favorable safety profile, with most adverse reactions being mild and reversible. Serious and persistent adverse reactions are rare, but long-term safety data beyond one year remain insufficient. Patient conditions should be evaluated and long-term monitoring conducted during treatment.

Current emerging clinical trials targeting the neuro-vascular-immune triad in rosacea are mostly single-center, small-sample designs with relatively short follow-up periods. There is insufficient data on long-term efficacy, relapse risk, and long-term safety. Some studies lack randomized double-blind placebo controls, leading to selection and assessment biases. The optimal dosage and treatment duration for various therapies have not yet been fully determined. However, existing research still holds significant value, providing innovative and evidence-based treatment options for rosacea. Future studies need to conduct multicenter, large-sample, randomized double-blind controlled trials, stratifying enrolled populations by age, disease severity, and comorbidities, and extending follow-up periods to clarify long-term prognosis. At the same time, in-depth analysis of the neuro-immune-skin triad interaction mechanisms should be pursued, combined with multi-omics screening for biomarkers. Combination therapy is an important direction for future exploration. Literature has confirmed that combining antidepressants, anti-CGRP drugs, and beta-blockers with conventional treatments such as metronidazole and doxycycline can produce synergistic effects. There are also exploratory cases of BoNT/A combined with BBL and pregabalin combined with duloxetine, preliminarily validating the feasibility of combination therapy. Future research can further explore combination strategies between novel therapies and traditional treatments, as well as among different novel therapies, to optimize treatment outcomes for refractory rosacea.

In summary, rosacea is a complex psychosomatic disease with a high risk of neuropsychiatric comorbidities and significant psychosocial burden (76). Traditional treatment approaches that focus solely on anti-inflammatory therapy often fail to correct deep systemic abnormalities, resulting in poor long-term outcomes and persistent impairment in patients’ quality of life. In clinical practice, dermatologists need to adopt the concept of total patient care and work closely with psychologists and psychiatrists to comprehensively assess patients’ physical symptoms, psychological status, and social functioning. This collaborative approach aims to develop individualized, integrated treatment plans that break the vicious cycle between skin inflammation and psychological stress, ultimately achieving long-term effective control of rosacea.
